# Heritable risk factors associated with language impairments

**DOI:** 10.1111/j.1601-183X.2006.00232.x

**Published:** 2007-02

**Authors:** J G Barry, I Yasin, D V M Bishop

**Affiliations:** Department of Experimental Psychology, University of Oxford South Parks Road, Oxford, UK

**Keywords:** Nonword repetition, parents, risk factors, specific language impairment

## Abstract

There is a strong genetic contribution to children’s language and literacy impairments. The aim of this study was to determine which aspects of the phenotype are familial by comparing 34 parents of probands with language/literacy impairments and 33 parents of typically developing probands. The parents responded to questionnaires regarding previous history for language/reading impairment and participated in psychometric testing. The psychometric test battery consisted of tests assessing non-verbal IQ, short-term memory, articulation, receptive grammar, reading abilities and spelling. Self-report measures demonstrated a higher prevalence of language and literacy impairments in parents of affected probands (32%) compared with parents of unaffected probands (6%). The two groups of parents differed significantly in their performance on the non-word repetition, oromotor and digit span tasks. Non-word repetition gave the best discrimination between the parent groups even when the data from the parents who actually were impaired as ascertained by direct testing or self-report were removed from the analyses. This suggests that non-word repetition serves as a marker of a family risk for language impairment. The paper concludes with a discussion of issues associated with ascertainment of specific language impairment (SLI).

Specific language impairment (SLI) is diagnosed when a child is significantly delayed in speech and language development despite having normal hearing, normal intelligence and no known neurological problems. Many years of research have yet to reveal all the factors that contribute to the expression of SLI; however, there is mounting evidence that the disorder is heritable (Bishop & Edmundson 1986; Bishop *et al*. 1996; Lewis & Freebairn 1992; Lewis *et al*. 2004; for review Stromswold 1998; Tallal *et al*. 1989a; Tomblin 1989). The discovery that a point mutation on a gene was associated with language impairment in three generations of a family, called the KE family (Lai *et al*. 2001), has led to a growing interest in identifying other genes involved in determining the course of language development for other phenotypes of the disorder (Bishop 2002; for review SLI Consortium 2002, 2004). Such research can be carried out either by looking at the same phenomena within families or by tracking down the genes or the loci of gene change that are correlated with observed differences in language development among individuals. The research endeavor has wide implications for understanding the biological basis for language and for understanding how genes and environment interact to affect different language learning outcomes. Further, if we can understand the role of genes in affecting language learning outcomes, the development of biologically based interventions to supplement more conventional methods of intervention becomes a possibility.

Research involving molecular genetics and/or pedigree analysis depends crucially on having good measures for the phenotype under investigation. An imprecise diagnosis of the phenotype can result in a genetically heterogeneous sample which will in turn significantly impact on the research (SLI Consortium 2004). Unfortunately, the SLI phenotype by its very nature is heterogeneous. The disorder is diagnosed on the basis of (i) a low score on a subset of language tests from a battery assessing receptive, expressive and phonological skills and (ii) no other impairment that could potentially explain poor performance (Bishop 2001). In making this diagnosis, different researchers use different language tests and different cutoffs. To complicate matters still further, the phenotype can vary with age, that is, a child may present with phonological impairments at a young age which may develop into expressive or literacy problems or may even resolve as the child matures.

The fact that the phenotype changes over time has important ramifications for distinguishing between affected and unaffected members of the same family. An affected relative may have had difficulties in the past which are completely resolved or may be impaired but on different tasks to the proband under investigation. It is thus an urgent research priority to discover which measures are most sensitive for detecting residual or subclinical language impairments in relatives of children with language or literacy impairments.

## Family aggregation studies, pedigree studies and self-report measures

Most of the data used in family aggregation/pedigree studies of SLI have come from questionnaire/interview materials (Bishop & Edmundson 1986; Lewis 1992; Lewis *et al*. 1993; Neils & Aram 1986; Tallal *et al*. 1989b; Tomblin 1989; Whitehurst *et al*. 1991). In these studies, parents were required to report on language and related problems in relatives of an affected child. All, with the exception of Whitehurst *et al*. (1991), report an increased prevalence of language or literacy impairment in families of affected children. Rates of affected first-degree relatives for probands with SLI vary from 77% (Tallal *et al*. 1989a) to 24% (Bishop & Edmundson 1986). A higher prevalence of affected fathers to mothers has also been reported with ratios varying from 1.6:1 (Lewis 1992) to 4.5:1 (Rice *et al*. 1998).

To a large extent, the variability in the rate of affected relatives depends on the specificity of the phenotype of the proband recruited to the study. For example, Lewis (1992) studied probands with phonological disorders, whereas Rice *et al*. (1998) focused on probands with a purely grammatical impairment.

The prevalence of language impairment among family members based on self-report data also depends on the strictness of the criteria used for determining whether a relative is affected or not. Tallal *et al*. (1989a) defined parents as affected where they reported a diagnosis of language impairment or where there was evidence of delayed reading and writing achievement or other poor performance at school. Using these criteria, they reported that 42% of first-degree relatives for the SLI proband had a positive family history for impairment. However, 19% of relatives in their control probands also provided evidence for some form of language delay. By contrast, Tomblin (1989) required that relatives must have received speech or language therapy as a child to be considered affected. He identified 19.57% of mothers and 19.05% of fathers of probands with language disorders as affected as compared with 4.51% of mothers and 3.31% of fathers of control probands.

Finally, rates of prevalence of the disorder in relatives depend on phenotype specificity in the study. Lewis (1992 demonstrated this effect in a study of nuclear family members of children with phonological disorders. She found that if only speech and language impairments were considered, 26% of first-degree relatives of language probands also had a history of SLI as against 4% of relatives of control probands. If dyslexia and other learning disabilities were included, the prevalence increased to 43.34% as compared with 9.05% in the parents of control probands (calculated from data provided in Lewis 1992).

## Direct testing of language abilities in adults

There is a paucity of tests available that have been normed for use with adults. In fact, apart from a few studies such as Lewis and Freebairn (1992, 1998) and Tomblin *et al*. (1992), there have been relatively few studies investigating how language difficulties manifest themselves in the adult population. Lewis and Freebairn (1992, 1998) were interested in understanding the residual effects on speech and language of adolescents and adults with a preschool history of phonological disorders. In addition to standard tests of language and literacy, they probed for evidence of residual impairments of the phonological system. Tomblin *et al*. (1992) on the other hand were interested in adults with a diagnosis of SLI, which included a broad range of impairments. Their battery aimed at investigating language abilities at the word and sentence levels and across a wide range of modalities: listening, speaking, reading and spelling.

Despite differences in test materials and participants’ speech and language histories, both groups of researchers found evidence for some form of residual impairment to the language system. Tomblin *et al*. (1992) further reported that language measures that placed high demands on information processing and phonological performance permitted a very accurate diagnosis of adults with a history of SLI. They found that a test battery probing spelling, speaking rate, sentence comprehension and receptive vocabulary abilities successfully placed the 70 people in their study into the appropriate diagnostic category with an error rate of 3%.

The same test battery has since been employed in a number of studies involving adults with language impairments (Clark & Plante 1998; Plante *et al*. 2001, 2002). Interestingly, Clark and Plante (1998), investigating brain morphology in language impairment, observed that performance on the test battery was a better predictor of ‘anomalous brain morphological features’ than classification using self-report. The data suggest a correlation with biological differences between affected and unaffected adults and provide independent evidence for the test battery’s validity as a tool for ascertainment of language status.

Finally, Plante *et al*. (1996) compared four methods for identifying adults affected by language impairment. Using self-report measures, between 12 and 37% of the parents of a language proband were classified as affected. Differences in rates of affection depended on diagnostic criteria, that is, whether participants had received speech and language therapy as a child (12.5%) or whether they had a history of language problems and/or academic difficulties including delays in reading or writing (37.5%). By comparison, behavioral measures identified 62% of parents of language probands as ‘affected’. Plante *et al*. (1996) concluded that case history underestimated the true rate of affected people in their test sample. However, behavioral testing also identified 16–25% of parents in the control proband as being affected. These latter findings suggest that the test battery may not be sufficiently specific to SLI. Some tests in the test battery focused on general literacy skills such as word knowledge and spelling ability. It is possible that performance on these tasks also reflected differences in literacy or environmental factors such as educational history.

## Non-word materials for testing for impairment

Tomblin *et al*. (1992) tested for residual language difficulties by placing heavy demands on participants’ language performance. An alternative strategy for finding residual impairments is to specifically target underlying cognitive deficits implicated in SLI.

SLI has been hypothesized to develop out of a deficit in phonological short-term memory (Gathercole & Baddeley 1990). This deficit is thought to impact on the children’s ability to learn new words and identify new syntactic structures. Phonological short-term memory capacity is tapped through tasks such as repetition of non-words like ‘blonterstaping’. The non-word repetition task gives participants minimal time to process the materials and they cannot draw on previous experience to help them complete the task. It is also largely independent of IQ (Bishop *et al*. 1996) and is relatively unaffected by social or ethnic background (Campbell *et al*. 1997). Finally, non-word repetition is thought to be a particularly good marker of a heritable phenotype because it is highly heritable and is also sensitive to residual problems in children with a past history of SLI (Bishop *et al*. 1996).

It is unclear whether the task will be sufficiently sensitive as a behavioral marker in adults, because to our knowledge, no study to date has investigated non-word repetition in parents of probands with SLI. The task has, however, been used in studies of parents of children with autism (Bishop *et al*. 2004). Many children with autism present with a similar profile of language difficulties as children with SLI, and Tager-Flusberg and Joseph (2003) have hypothesized that the same genes are implicated in the two disorders. Bishop *et al*. (2004) found that although children with autism were impaired on the non-word repetition task, there was no relationship between the scores obtained by these children and the scores obtained by their nuclear family relatives. This stands in stark contrast with patterns of performance observed in twins with SLI (Bishop *et al*. 1996). It is therefore of considerable interest to investigate whether parents of children with SLI exhibit deficits on this task.

In summary, self-report has been used to investigate the heritability of SLI, but results are variable. Behavioral testing is also problematic and care is needed in selecting appropriate tasks that minimize the risk of over/under-identifying cases of language impairment. Our aim is to determine the most appropriate methods for discriminating between affected and non-affected family members by addressing the following hypotheses:

1. Parents of probands with language/literacy impairments will report a higher rate of familial language impairment than parents of typically developing probands
2. Direct tests of language and literacy skills will reveal a higher prevalence of communication problems in parents of affected children than self-report
3. Tests using nonsense materials, that is, non-word repetition and non-word reading will be particularly good markers of familial status.

As discussed, the phenotype of SLI can vary over time and there is some doubt about how valid it is to specify a narrow phenotype (Kamhi & Catts 1986; Lewis & Freebairn 1998; Lewis *et al*. 2004). When establishing our research questions, we therefore chose to permit probands with a broad range of language and/or literacy impairments.

## Materials and methods

### Participants

All participants were parents of probands who were taking part in a study of the causes and correlates of language/literacy impairments. They were recruited into this study at the time that they signed consent for their child(ren) to participate. Probands with language/literacy impairments (*n* = 30) were recruited either through schools offering support to children with speech and language difficulties or by advertising in the newsletter of Afasic, a UK-based charity providing support and information networks for families with children with communication difficulties. Typically, developing probands (*n* = 33) were recruited through local primary and secondary schools.

To participate, parents had to be biological parents of the probands and have normal hearing (a bilateral pure tone audiometric screening test at 25 dB HL ISO for 500, 1000 and 2000 Hz), a non-verbal IQ (NVIQ) of 80 or above on the Wechsler Abbreviated Scale of Intelligence (WASI) (Wechsler & Chen 1999), English as a mother-tongue and no reported neurological disorders. Parents of probands with language/literacy impairments were recruited only if their child met the following criteria: WASI non-verbal IQ of 80 or above and performance below the 10th centile on at least two standardized tests of language or literacy ability (Table 1). Although all children had been recruited as having oral language difficulties, to be included as a proband, it was sufficient that they failed two tests from the battery. For 3 of the 30 probands this criterion was met for literacy measures only. Typically developing probands had no history of language difficulties and no more than one language measure below the 10th centile. We included control children who had a single low score because in a lengthy battery with 11 test measures, we have found it is not unusual to obtain the occasional low score; this was the case for 6 of our 33 controls, none of whom was thought to have any language or educational difficulties.

**Table 1:** Tests used to select probands and mean and standard deviation (SD) scores for probands with language/literacy impairments and control probands

Test	Language/literacymean (*n* = 30)	SD	Controlmean (*n* = 33)	SD
Non-verbal IQ	98.1	8.5	103.0	11.0
Test for reception of grammar-2 (Bishop 2003b)*	89.1	13.4	102.2	6.8
Expression, reception and recall of narrative instrument (Bishop 2004)*				
Initial story-telling	93.7	13.6	100.8	9.4
Story recall	92.2	18.0	103.8	9.8
Forgetting score	94.8	16.4	103.3	9.3
Comprehension	94.2	19.1	106.3	14.6
Mean length of utterance (MLU)	94.4	13.9	101.0	15.2
NEPSY (Korkman *et al*. 1998)†				
Repetition of nonsense words	7.6	3.0	11.6	2.2
Sentence repetition	5.3	2.5	11.2	2.9
Test of word reading efficiency (Torgesen *et al*. 1999)*				
Sight word efficiency	80.4	14.5	98.7	13.0
Phonemic decoding efficiency	77.8	13.4	108.7	10.5
Children’s communication checklist-2 (Bishop 2003a)‡				
Global communication composite	35.8	14.4	77.1	19.7

NEPSY, NEuroPSYchology; TOWRE, test of word reading efficiency.

Nine of the language and literacy probands met inclusion criteria on the basis of the language tasks; three met inclusion criteria on the TOWRE subtests; 18 had language and literacy deficits.

* Normative mean = 100, SD = 15.

† Normative mean = 10, SD = 3.

‡ Cutoff for 10th centile = 55.

Demographic details for participating parents are summarized in Table 2. It is evident without statistical testing that the two groups are well matched in gender ratio, age, NVIQ and educational level.

**Table 2:** Demographic details for the two groups of participating parents

	Proband status	
	
	Control(*n* = 33)	Language/literacy (*n* = 34)
	(couples = 0)	(couples = 4)
Sex	7 M, 26 F	8 M, 26 F
Age (years)		
Mean	43.8	43.5
SD	5.4	5.3
Range	34.8–56.3	34.0–56.1
WASI NVIQ		
Mean	112.5	111.4
SD	11.0	13.7
range	92–141	85–138
Age at leaving full-time education (years)*		
Mean	19.2	18.7
SD	2.8	2.8
Range	15–26	15–24

* Some parents went to university/college to study as mature-age students. The number of years spent studying was added to the age at which they first left school.

### Procedure

#### Psychometric assessment battery for parents

The assessment battery consisted of 10 psychometric tests and a hearing screen. It took, on average, 45 min to administer. The battery included tests of non-verbal reasoning, short-term memory, understanding of grammar, reading skills, spelling, oromotor coordination and phonological short-term memory. Where indicated below, some tests were presented through wav files on a DELL Latitude D800 computer with a built-in sound card (20-bit Sigmatel audio-sound card), through Sennheiser HD 25-1 headphones that attenuated external noise by 40 dB SPL.

The block design and matrix reasoning task from the WASI (Wechsler & Chen 1999) were used to assess non-verbal reasoning skills. Scores were converted into age-scaled scores.

Short-term memory was tested using a digit span task. This task was a modified version of the digits forward condition of the digit span task from the Wechsler Intelligence Scale for Children (WISC-R) assessment battery (Wechsler 1974). Participants were required to repeat a list of numbers that were read out to them at a rate of one digit per second. The lists consisted of between two and nine digits which were presented in order from shortest to longest with two trials at each list length. Each correctly repeated series of numbers was awarded a score of 1. The data are presented as raw scores, that is, number of correctly repeated lists with a maximum possible raw score of 16.

The electronic version of Test for Reception Of Grammar-2 (TROG-2 Bishop 2003b) was used to assess receptive grammatical knowledge. Participants heard a sentence played over headphones and were asked to identify which of four pictures corresponded to the sentence they had just heard. Each grammatical construction was presented in a block of four. To pass a block, participants had to correctly identify all four presentations of the construction. Raw scores (number of blocks correct) were converted into centiles and scaled scores using norms derived from British adults.

Reading skills were assessed using form B of the Test Of Word Reading Efficiency (TOWRE) (Torgesen *et al*. 1999). Here, participants are required to read two lists of items as fast and accurately as possible within a 45-second period. The first list (sight word reading efficiency) contains real words, and the second list (phonemic decoding efficiency) contains non-words, for example, ‘revignuf’. The test was scored on-line and also recorded so that scoring could be rechecked. Raw scores were converted to scaled scores using American norms for adult readers.

The spelling task was designed for this study and consisted of 40 words, some of which had regular spellings, for example, ‘bed’ and some of which were uncommonly occurring or were irregular, for example, ‘yacht’ or ‘rhyme’. The task was to write as many dictated words as possible in a two-minute period. The data are summarized as raw scores with a maximum score of 40 words correct.

The NEuroPSYchology (NEPSY) test battery (Korkman *et al*. 1998) was developed for assessing neuropsychological development in children aged from 3:0 to 12:11 years. Two tasks were included in the adult assessment battery: the oromotor sequences task and the repetition of nonsense words (non-word repetition) task. Since norms for the tasks do not extend to the adult population, raw scores are reported.

The oromotor sequences task assesses oromotor coordination ability. This skill is thought to be important for the smooth production of sequential speech sounds. Early items in this task require speakers to repeat a series of difficult to articulate sound sequences, for example, ‘squish squash’ and a series of tongue-twisters, for example, ‘red leather, yellow leather’ five times. Later items involve repeating short sentences that may also tax short-term memory skills, for example, ‘Put the pepper beads in the paper bag’. The maximum raw score for this task was 70.

The non-word repetition task consisted of 13 nonsense words ranging in length from two to five syllables which were prerecorded using a female standard Southern British English accent. The non-words were digitized and presented from a computer through headphones. Responses were scored on-line and also recorded for rechecking. Each correctly produced syllable was given a score of 1. The maximum possible score for this task was 46.

#### Self-report measures

Each participant completed a questionnaire which was designed to elicit information about their educational, medical, and speech, language and literacy histories.

Based on their proband and on their responses to questions regarding family history, participants were categorized as having (i) no family history of language/literacy impairment; (ii) a marginal family history (i.e., anecdotal problems in the family which were unsubstantiated by testing) or (iii) a clear family history, that is, child/family member/or personal history of difficulties with speech, language or literacy. Participants were then also categorized according to whether they had a first-degree relative (excluding children) who had experienced difficulties with speech or language.

## Results

### Summary of family history of language impairment

By definition, all parents of affected probands had a family history for language/literacy impairment. Eight parents (23.5%), however, also had an additional first-degree relative with a history of language or literacy impairment. Of the parents of typically developing probands, four out of 33 (12%) had a marginal family history for language impairment. One parent reported having a brother who stuttered and three parents reported that a child not recruited to the study had a language or literacy delay (two children were dyslexic and one had a semantic-pragmatic language impairment). Data were missing for three parents (one in the control group).

### Hypothesis 1: parents of probands with language/literacy impairments will report a higher rate of familial language impairment than parents of typically developing probands

Thirteen parents reported having a personal history of language difficulties. Eleven of these (32.4%) were parents of probands with language/literacy impairments as compared to 2 (6%) who were parents of typically developing probands. These proportions are significantly different on a Fisher Exact Test one-tailed *P* = 0.007. The data thus supported previous research and our first prediction of a higher prevalence of language or literacy difficulty among parents of affected children.

Of the two affected parents who were related to typically developing probands, one received speech and language therapy when she was younger, the other reported on-going problems with indistinct speech as well as difficulties with reading and writing. The affected parents of the probands with language/literacy impairments varied considerably in type and severity of difficulty reported. Most parents reported a history of delayed reading, and six said they still experienced some form of difficulty with speech, language or reading.

Proportionately more males than females had a history of language impairment. This is particularly striking in the language/literacy group where six out of eight men (75%) vs. five out of 26 (20%) women had a history of language impairment. The data correspond well with data from studies of Rice *et al*. (1998) and Tomblin (1989) who also observed a higher prevalence of language impairment among fathers of language probands.

We used a volunteer group of participants. Far fewer fathers than mothers took part. It is possible that we overestimated language impairment if a general reluctance of males to participate was overcome by interest in the study of those with a personal history of language impairment. We have anecdotal evidence both for and against this as a possible explanation of our results.

### Hypothesis 2: direct tests of language and literacy skills will reveal a higher prevalence of communication problems in parents of affected children than self-report

Children were required to perform below the 10th centile on two or more tasks to be identified as affected by language or literacy impairments. When the same criterion was applied to the adult data, we found that eight parents of a language/literacy proband (24%) and three parents of a control proband (9%) were identified as affected. This was non-significant (Fisher Exact test one-tailed *P* = 0.102). This finding contrasts with the strong association found between self-report and proband status and suggests that direct testing was less sensitive than self-report for identifying affected parents. Table 3 comprises two 2 × 2 tables summarizing numbers of parents diagnosed as affected using direct testing and numbers diagnosed by self-report. However, if we use the frequencies of self-reported language impairment in the two groups to derive expected frequencies, and test the frequencies obtained using direct tests of language, the difference between observed and expected frequencies is not significant in a chi-square test; χ^2^ = 1.74, df = 1, *P* = 0.18. Thus, although self-report gives a significant association between parent and proband status and direct testing does not, the difference between the two methods is not statistically significant in this sample.

**Table 3:** A summary of the numbers of parents in each proband group that were diagnosed as affected using either direct tests or self-report

	Language/literacyself-report		Controlself-report	
		
Direcr test	+	−	+	−
+	5	3	1	2
−	6	20	1	29

### Hypothesis 3: Tests using nonsense materials will be good markers for familial status

The data reported so far do not provide compelling evidence in support of direct testing as method of ascertainment. However, the method was evaluated using cutoff scores in a battery of tests, and group differences may have been diluted by including insensitive measures. The next set of analyses looked at the sensitivity of individual tests for discriminating between parent groups. Performance on these tests is summarized in Table 4. Quantitative differences between the two groups of parents were explored using a manova.

**Table 4:** Mean and SD scores on language and literacy measures for the two groups of parents

	Proband status						
		
	Control			Language/Literacy			
	Mean	SD	n*	Mean	SD	n*	*T*-tests
Oromotor (raw out of 70)	64.0	4.6	4	59.0	5.0	16	*t* = − 4.163, *P* < 0.001
Nonword repetition (raw out of 46)	41.0	3.9	3	36.8	4.4	6	*t* = − 4.063, *P* < 0.001
Digit span (raw out of 16)	11.1	2.2	1	9.6	2.1	9	*t* = − 2.869, *P* < 0.01
TOWRE sight words (scaled)	93.2	12.0	4	90.2	16.2	7	*t* = −0.674, ns
TOWRE phonemic decoding (scaled)	99.1	12.9	2	92.5	16.5	4	*t* = − 1.574, ns
Spelling task (raw out of 40)	36.5	4.1	3	34.1	7.7	5	*t* = − 1.496, ns
TROG-2 (scaled)	101.8	7.9	1	99.5	7.8	0	*t* = − 1.188, ns

* *n* refers to parents scoring below the 10th centile.

Before submitting the data to the manova, univariate normality for each of the dependent variables was tested (Kolmogorov–Smirnov test of normality, *P* > 0.05). Scores for the oromotor (raw), word reading (scaled) and digit span (raw) tasks were normally distributed within the two groups of participants. Scores were non-normally distributed among the parents of affected probands for non-word reading and non-word repetition. They were non-normally distributed among both groups for TROG-2 and spelling. To normalize the distributions on the spelling task, the raw data were converted to stanines (Guildford & Fruchter 1973). Most parents performed at ceiling on TROG-2, and the task was consequently excluded from further analysis.

The data entered into the manova satisfied the requirements for homogeneity of covariance matrices [Box’s test, non-significant (ns), *P* = 0.340] and equality of variances for each dependent variable (Levene’s tests, ns). The analysis indicated a significant difference between the two groups on the test battery (Exact *F*_5,59_ = 5.00, *P* < 0.001, Pillai’s trace test statistic). Follow-up univariate tests showed a significant effect at the 0.01 level for the oromotor, non-word repetition and digit span tasks, with effect sizes (η^2^) of 0.204, 0.205 and 0.109, respectively.

To determine which subset of tests best discriminated between the two groups of parents, the six measures were entered into a discriminant analysis. Canonical variate correlation coefficients of 0.712 and 0.710 were obtained for the non-word repetition and oromotor tasks, respectively, suggesting that both tasks were similarly successful at discriminating between the two groups of participants. However, only one variable (non-word repetition) was required to explain 100% variance between the two groups.

Finally, the one-variable model with non-word repetition as the predictor variable was tested for its success in classifying parents according to proband status. Of the 66 participants included in the model (one participant from the language/literacy group was excluded due to missing data), 75.8% were correctly classified by it: 24 participants in the language/literacy group and 26 in the control group. This gives a specificity (% unaffected individuals correctly classified) of 78.7% and a sensitivity (% affected individuals correctly classified) of 70.1%. These rates of specificity and sensitivity are poor compared with respective rates of 100 and 97% reported by Tomblin *et al*. (1992). The differences reflect differences in methodology. The parents in this study were recruited on the basis of the proband’s language status, not their own. By contrast, all the participants in the Tomblin *et al*. (1992) study had a history of language impairment.

### Do ‘unaffected’ parents of language/literacy probands differ from unaffected parents of control probands?

The group differences summarized in Table 4 suggest subtle deficits in the parents of affected children. Alternatively, they may simply be due to the inclusion in the language/literacy group of more parents who were themselves affected. To distinguish between these possibilities, the analyses were repeated, this time excluding all parents self-reporting language/literacy problems and four parents in the control group who were classified as having a marginal family history. The resulting groups are referred to as Ctrl-SR and LL-SR to denote parents of control and language/literacy probands who do not self-report a history of language problems.

All the tasks were normally distributed except the stanines for the spelling task for the Ctrl-SR group and standard scores for non-word reading for the LL-SR group. The data satisfied the criterion for equality of covariance of matrices (Box’s test, *P* = 0.284, ns) and for equality of variances for each of the dependent variables (Levene’s tests, ns). The data were submitted to a manova and a significant difference between the two groups was found (Exact *F*_5,42_ = 3.743, *P* < 0.01, Pillai’s trace test statistic). There was a significant effect at the .01 level for the oromotor and non-word repetition tasks and at the 0.05 level for the digit span task (η^2^ = 0.134, 0.240, 0.079, respectively). Discriminant analysis indicated that only one factor, non-word repetition, was required to discriminate between the two groups. This task correctly classified 75.5% of participants in the two groups.

An even more stringent test involves excluding from analysis the 11 parents who were affected by language impairment as indicated by direct testing. When the remaining data were entered into a manova, a significant difference between the two groups was found once more (Exact *F*_5,48_ = 3.164, *P* < 0.05, Pillai’s trace test statistic), with significant effects at the 0.01 level for the oromotor and non-word repetition tasks only (η^2^ = 0.141, 0.203, respectively). Discriminant analysis again indicated that non-word repetition was the only factor required to discriminate between the two groups with 70.9% of the two groups being correctly classified by the task. This analysis thus demonstrated a deficit in non-word repetition in parents of language probands even when they did not meet our criteria for impairment.

### Is there a difference between those self-reporting difficulties with language and those reporting difficulty primarily with reading?

For a final analysis, the status of the children was ignored and we focused instead on the phenotype in the adults with language or literacy deficits as measured by self-report and direct testing.

Thirteen parents had a history of language or literacy impairment. Eight of these (SR-read) described themselves as slow readers or had been diagnosed as dyslexic. The remaining five parents (SR-lang) self-reported a history of speech and language impairments with all except one also reporting difficulty with reading. Independent *t*-tests indicated that the SR-lang group was significantly worse than the SR-read group on non-word repetition (*t* = 2.915, *P* < 0.05). Otherwise, there were no significant differences between the two groups on any of the other tasks in the battery. The numbers, however, for each group are not large which impacts on the power of the analyses to find an effect. To clarify that this result is not due to the performance of a couple of individuals, the data for each parent in the self-report group were ordered according to performance on non-word repetition Table 5). Group data for the remaining parents of both proband groups were also included for ease of comparison.

**Table 5:** Individual data for parents self-reporting difficulties with language or literacy skills

Phenotype	L	L	R	L*	L	L*	R	R	R	R	R	R	R	LL-SR	Ctrl-SR
*N*-test low	5	3	5	2	1	1	1	1	2	0	0	1	0	–	–
NW-read	*55*	*55*	*55*	89	98	91	81	98	*76*	112	109	89	81	99.4 (11.7)	100.0 (11.8)
Spell	*7*	35	*12*	35	37	34	*24*	39	34	38	38	*25*	38	36.9 (3.1)	36.9 (4.0)
Oromotor	*50*	*58*	*45*	70	59	60	60	*58*	60	59	*56*	*57*	*58*	60.5 (4.7)	64.2 (4.4)
Digit span	*6*	8	*5*	9	10	9	12	11	*6*	9	11	9	13	9.8 (1.8)	11.0 (2.1)
NW-rep	*25*	*26*	*28*	*31*	*33*	*34*	35	36	38	38	38	40	44	38.0 (2.9)	41.4 (3.3)

Ctrl-SR, parents of control probands not self-reporting difficulties; L, SR-lang; LL-SR, parents of language/literacy probands not self-reporting difficulties; *N*-test low, number of tests below the 10th centile; R, SR-read.

The data are organized according to performance on the non-word repetition task (lowest to highest). Scores below the 10th centile are in italics. Standard Deviations are in parentheses.

* Parent of control proband: all other individual cases are parents of language/literacy probands.

All members of the SR-lang group had non-word repetition scores below the 10th centile (i.e., <35) unlike the SR-read group who presented with raw scores for non-word repetition ranging from 28 to 44. The ordering of the data in Table 5 suggests a phenotypic spilt based on non-word repetition. A hierarchy of performance on non-word repetition from worst to best performance can be mapped out as follows: SR-lang < SR-read ≈ LL-SR < Ctrl-SR

To verify that the effect was still present for the 11 parents identified as having language and/or literacy impairments using direct testing, a similar table was mapped out for this group (Table 6). Where available, diagnoses based on self-report were included in the table as a prefix ‘SR’. Three parents performed below the 10th centile on the tasks assessing literacy skills alone, and it was not possible to compare group means. However, a similar split in phenotype based on non-word repetition ability is suggested by the data.

**Table 6:** Individual data for parents who scored below the 10th centile on two or more tasks

Phenotype	^SR^L/L	^SR^L/L	^SR^R/L	^SR^L/L*	L	L	L	R*	^SR^R/R	^SR^R/R	L*	LL-DT	Ctrl-DT
Word-read	*56*	83	*64*	90	85	87	*77*	86	85	*71*	93	95.3 (14.5)	93.8 (11.9)
NW-read	*55*	*55*	*55*	89	92	95	86	*77*	*76*	89	*77*	98.9 (12.0)	100.9 (11.9)
Spell	*7*	35	*12*	35	36	*28*	32	*25*	34	*25*	31	36.8 (3.6)	37.2 (3.5)
Oromotor	*50*	*58*	*45*	70	*53*	*55*	*55*	64	60	*57*	*54*	60.9 (3.9)	64.2 (4.3)
Digit span	*6*	8	*5*	9	8	9	*6*	*7*	*6*	9	14	10.4 (1.6)	11.2 (2.1)
NW-rep	*25*	*26*	*28*	*31*	*31*	*34*	35	37	38	40	40	38.4 (2.6)	41.5 (3.5)

Ctrl-DT, unaffected parents of control probands; L, poor performance on language and literacy tests; LL-DT, unaffected parents of probands with language/literacy deficits; R, poor performance on literacy tasks only; ^SR^L, self-report language/literacy impairments; ^SR^R, self-report literacy impairments.

The data are organized according to performance on the non-word repetition task (lowest to highest). Scores below the 10th centile are in italics. Standard Deviations are in parentheses.

* Parent of control proband: all other individual cases are parents of language/literacy probands.

## Discussion

As a first step toward finding tests that were sensitive to familial language impairment, we verified using self-report data that there was indeed a higher prevalence of impairment among the parents of probands with language/literacy impairments. In the case of all but four probands, only one parent (typically the mother) participated in the study. Even so, self-report measures confirmed a strong pattern of familial association with language/literacy impairment. Prevalence rates were 32% among parents of language probands and 6% among parents of control probands. These rates were in the range reported for first-degree relatives by Bishop and Edmundson (1986) and Neils and Aram (1986), that is, from 24 to 42% for language probands and 3–8% for control probands. The data suggested that we had recruited a fairly typical group of parents to the study.

We then compared the prevalence of language/literacy impairments as measured by self-report with the prevalence as measured by direct testing. Plante *et al*. (1996) found that case history/self-report measures significantly underestimated the numbers of affected parents of language probands compared with behavioral measures. We too predicted a higher prevalence of impairment using behavioral measures. Our hypothesis was not supported by the data. Thirteen of 67 parents were identified as being affected using self-report measures compared with 11 using direct tests. When we separated the data according to proband status and compared results from self-report and direct testing, a trend was observed for the parents of language/literacy probands in favor of self-report as a method of ascertainment. Tallal *et al*. (2001) and Conti-Ramsden *et al*. (2006) also compared rates of language impairment ascertained using direct testing with rates ascertained using self-report data. Both studies reported that the two methods of ascertainment yielded similar rates of prevalence. Tallal *et al*. (2001) further noted that there was only 74% agreement between self-report and direct testing. Where there was disagreement between the two methods, they found that self-report/family history data typically indicated the presence of a language impairment which was not subsequently supported by direct testing. We too did not observe a high rate of agreement between the two methods of ascertainment and like Tallal *et al.* we observed a trend in favor of self-report, that is, only six out of 13 parents identified by self-report also met criteria for language/literacy impairment by direct testing. Notably, we had a lower rate of agreement between the two methods of ascertainment than Tallal *et al*. (46 vs. 74%). This may reflect differences in test battery, Tallal *et al*. (2001) used the token test employed by Tomblin *et al*. (1992) in their adult test battery, in combination with a language test battery for use with adolescents. Alternatively, the differences in rates of agreement between direct testing and self-report observed in the two studies may reflect differences in definition of impaired performance. In this study, we defined impaired performance as being below the 10th centile (1.27 SD). This is more stringent than the criterion applied by Tallal *et al*. of performance below 1 SD.

Given the low level of agreement between direct testing and self-report, a question arises regarding the preferred method for determining prevalence of language impairment. We will leave discussion of this until after reviewing the results from direct testing.

### Tests that are familial with language and literacy impairments

The focus of this research was on finding behavioral tests that would reliably identify adults affected by language impairment. Core to the development of the test battery was the idea that an underlying cognitive deficit was implicated in the expression of language/literacy impairments. We hypothesized that this deficit would be revealed through tasks involving nonsense materials such as non-word repetition and non-word reading. In fact, the groups only differed on the non-word repetition, oromotor and digit span tasks with no significant differences being observed on non-word reading. Discriminant analysis further revealed that performance on non-word repetition alone was able to correctly classify 75% of the participating parents into their proband groups. This effect remained even after excluding all parents identified as having a language impairment. The data further indicated a hierarchy of ability to perform the non-word repetition task such that affected parents were worse at the task than unaffected parents of probands with language/literacy impairments, who in turn were worse than parents of typically developing probands (Tables 5 and 6).

Our interest in the non-word repetition task stemmed from findings by Bishop *et al*. (1996) that twins with SLI had deficits in performing the task even after the outward manifestations of their language impairments had resolved. Bishop *et al*. further determined that the same genetic factors leading to the overt expression of SLI also led to deficits in non-word repetition. Our finding that deficits on this task are present in parents of probands with language/literacy impairments even in the absence of a personal history for language impairment provides further evidence for a heritable risk factor for SLI.

We expected significant differences in performance on non-word reading between the two groups of parents since the test battery used for proband ascertainment included tests for literacy and children with SLI have been reported to have phoneme decoding deficits (Briscoe *et al*. 2000; Stothard *et al*. 1998). Furthermore, familial aggregation studies of the dyslexia phenotype report a genetic basis for phonemic decoding skills (Raskind *et al*. 2000). As Table 5 illustrates, when the data for the parents reporting a history of language impairment were removed from the group data, the performance of the ‘unaffected’ parents of language/literacy probands was indistinguishable from that of the parents of typically developing probands. Both non-word repetition and non-word reading require some level of phonological processing; however, non-word repetition specifically probes phonological short-term memory, whereas non-word reading focuses on the ability to map between graphemes and phonemes. The fact that the two groups of ‘unaffected’ parents were virtually indistinguishable on the non-word reading task (Tables 5 and 6) suggests that a deficit in the latter process is not an underlying risk factor for SLI. By contrast, there is mounting evidence that phonological short-term memory is, and in this regard, it is noteworthy that both Kamhi and Catts (1986) and Goulandris *et al*. (2000) reported that children with SLI were worse at non-word repetition than children with reading deficits.

The oromotor task was almost as effective as the non-word repetition task in discriminating between the two groups of parents. Lewis and Freebairn (1992, 1998) made similar observations regarding first-degree relatives of children with phonological disorders. The question is, have output problems resulted in the differences observed in performance on non-word repetition?

Output problems have been implicated in non-word repetition ability even when, as here, participants do not have overt articulation problems (Bishop *et al*. 1996). Unfortunately, the oromotor task included in our test battery was not a pure test of oromotor skills since it also included tongue-twisters which taxed short-term memory. It is thus difficult to precisely state the extent to which output problems directly impacted on non-word repetition in our study. What we can say is that using a different array of tasks, Lewis and Freebairn (1992, 1998) also found a deficit in oromotor skills in first-degree relatives of children with phonological deficits. They referred to this deficit as a ‘verbal trait deficit’. It is possible that the oromotor and non-word repetition tasks are assessing two separate but not mutually exclusive underlying cognitive abilities. One system, tapped by non-word repetition, comprises phonological short-term memory while the second, tapped by the oromotor task, is associated with speech motor abilities. These two subsystems may interact to contribute to the development of speech and language learning disorders.

### Direct testing vs. self-report for ascertainment of language/literacy impairments

Apart from the test batteries developed by Tomblin *et al*. (1992) and Lewis and Freebairn (1992, 1998), there are no suitable test batteries available for use with adult populations. Certainly, there is no test battery available which includes a gold standard test for the diagnosis of SLI. We are thus reliant on self-report/case history material as a first step toward identifying people affected by the disorder. However self-report measures are not ideal since the data are based on retrospective information, and while they potentially provide access to valuable information about impairments that may have resolved, the data must also be treated with caution. Typically, self-report/case history materials provide little quantitative information about the severity or nature of the impairment in the past. Depending on educational experience and government policy at the time, adults may or may not have been identified as having a language impairment in their childhood. Finally, parents may over- or under-report a history of impairment for a variety of reasons including their personal level of awareness of the disorder.

The reliability of using direct testing alone for diagnosing SLI must also be questioned since the outward manifestations of the disorder can vary considerably with age, making it difficult to find tests that will target appropriate symptoms. The non-word repetition task is interesting because it seems to go beyond surface symptoms to tap into an underlying cognitive deficit. Moreover, from our research, it seems that not only are affected parents poor at the task but ‘unaffected’ parents of probands with language/literacy impairments also have deficits on the task – though at a subclinical level. In other words, many parents of language/literacy probands carry a heritable risk factor for SLI. Finally, as Catts *et al*. (2005) demonstrated, deficits in non-word repetition are not common to all phenotypic variants subsumed under the umbrella term ‘SLI’. From the perspective of ascertainment, if the appropriate range of tests is not included in a test battery then we run the risk of misdiagnosing participants which in turn will impact on research outcomes from studies investigating, for example, the molecular genetics of the disorder.

In sum, SLI is probably heterogeneous and the term may even prove a cover term for a number of different disorders. Until we fully understand how a genotype interacts with the environment to lead to phenotypic variants, we are limited in our ability to identify a test to serve as a ‘gold standard’ for the diagnosis of ‘SLI’. Self-report and case history provide different, but complementary information about a participant’s speech, language and literacy experience and without a gold standard test for SLI both measures have a valuable role to play in ascertaining the presence of language or literacy impairments.
